# Case report: Brain infarction following percutaneous drainage of a liver abscess post-chemotherapy for pancreatic head cancer

**DOI:** 10.3389/fmed.2024.1453533

**Published:** 2024-12-11

**Authors:** Wan-qiu Deng, Bo Wei, Zheng-yan Li, Kai Liu

**Affiliations:** ^1^Center of Infectious Diseases, West China Hospital, Sichuan University, Chengdu, China; ^2^Department of Gastroenterology, West China Hospital, Sichuan University, Chengdu, China; ^3^Department of Radiology, West China Hospital, Sichuan University, Chengdu, China

**Keywords:** chemotherapy, liver abscess, percutaneous drainage, brain infarction, pancreatic head cancer

## Abstract

Bacterial liver abscesses commonly occur in patients with immune deficiencies such as diabetes, post-chemotherapy, or post-immunosuppressive therapy. The recommended treatment for liver abscesses exceeding 5 cm in a diameter is anti-infection therapy combined with percutaneous catheter drainage. Complications may include local spread to adjacent tissues or organs and thrombosis of the liver and portal veins. Rare hematogenous spread to distant sites, such as endophthalmitis and central nervous system embolism, have been reported, though such complications are uncommon post-drainage. This case report details a patient who suffered a brain infarction shortly after percutaneous drainage of a large liver abscess.

## Introduction

Liver abscess refers to purulent lesions of liver tissue, which are caused by infection by various purulent microorganisms. It is clinically categorized into three types: bacterial, fungal, and amoebic, with bacterial liver abscesses being the most common. The disease occurs mainly due to the invasion of the liver by purulent bacteria, which can adversely affect some organs, with necrosis of the liver and liquefaction of abscesses—a potentially life-threatening disease characterized by the formation of a space-occupying lesion in the liver parenchyma. With the rapid development of the economy and the aging of the population, the incidence of metabolic diseases has been rising, resulting in an increase in the number of patients with PLA in combination with underlying diseases, corresponding to which the etiology, diagnosis, and treatment of PLA have undergone significant changes. The clinic is faced with new challenges. If PLA is not diagnosed in time and treated appropriately, it will lead to severe complications such as sepsis and infectious shock, and the mortality rate is as high as 10% ~ 30% (1). Traditional open treatment methods are usually used as insurance treatment options, and the preferred surgical option for patients is minimally invasive techniques. Ultrasound-guided puncture therapy is a relatively standard treatment measure, and the advantages of ultrasound intervention are more significant, with minimally invasive, shorter procedure times, milder patient stress, and a high degree of safety, which promotes postoperative recovery and ensures patient outcomes. Despite the current advances in diagnostic imaging and antibiotic therapy, the common complications of ultrasound-guided percutaneous drainage include poor catheter drainage, bleeding, infection, biliary fistula, extravasation of injected medication, and liver rupture, etc. (2). There may be a greater surgical risk for immunodeficient patients who are comorbid with a variety of underlying diseases. In this paper, we report a case of a large liver abscess that caused a brain infarction shortly after percutaneous drainage.

## Case presentation

**Figure 1:**
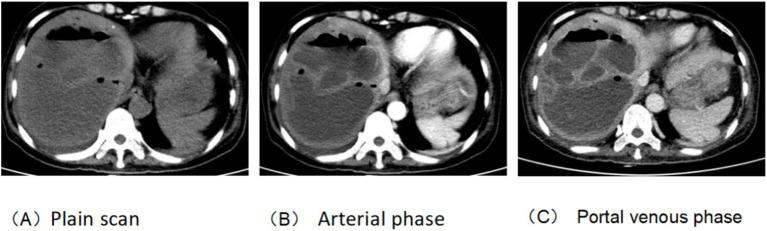
Upper abdominal enhanced CT. **(A)** Plain scan, large low-density shadows in the right and left inner lobes of the liver, Size about 14x10cm; there is separation and gas accumulation inside; **(B,C)** the lesion wall and septum were enhanced, which was considered liver abscess.

**Figure 2:**
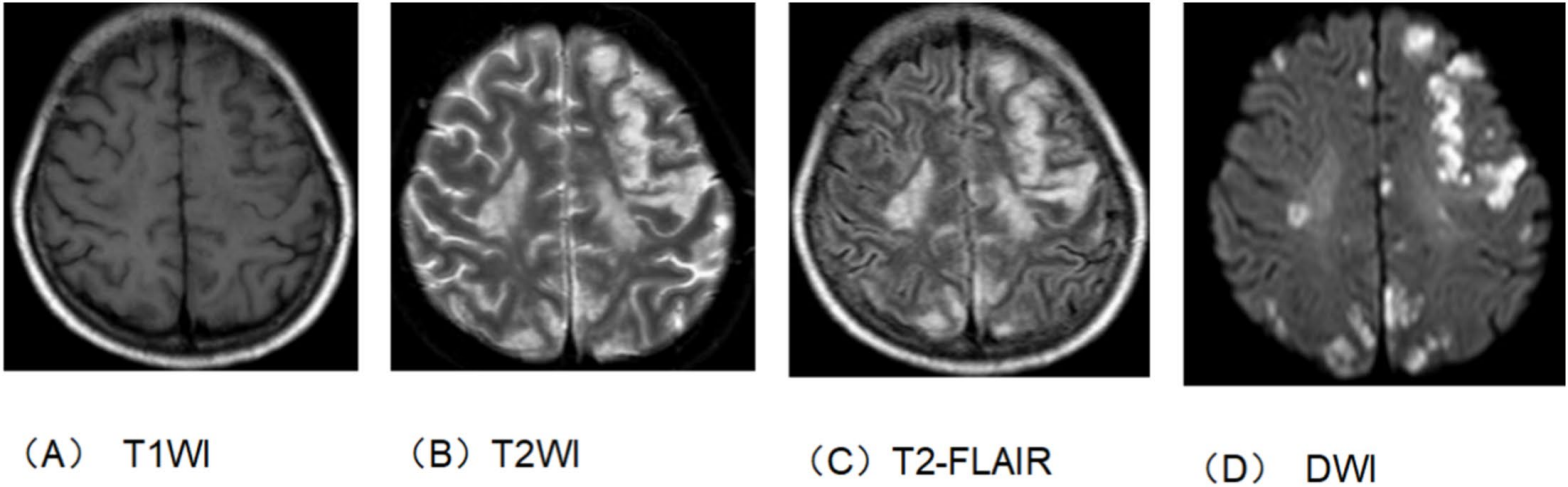
Head and neck enhanced MRI. **(A)** T1 sequence. **(B)** T2 sequence. **(C)** T2-flair sequence. **(D)** DWI sequence. The bilateral rated lobes scattered in multiple patchy and nodular slightly longer T1 and long T2 signal shadow. T2-flair showed hypertension and DWI showed high signal intensity. This suggests brain infarction.

**Figure 3:**
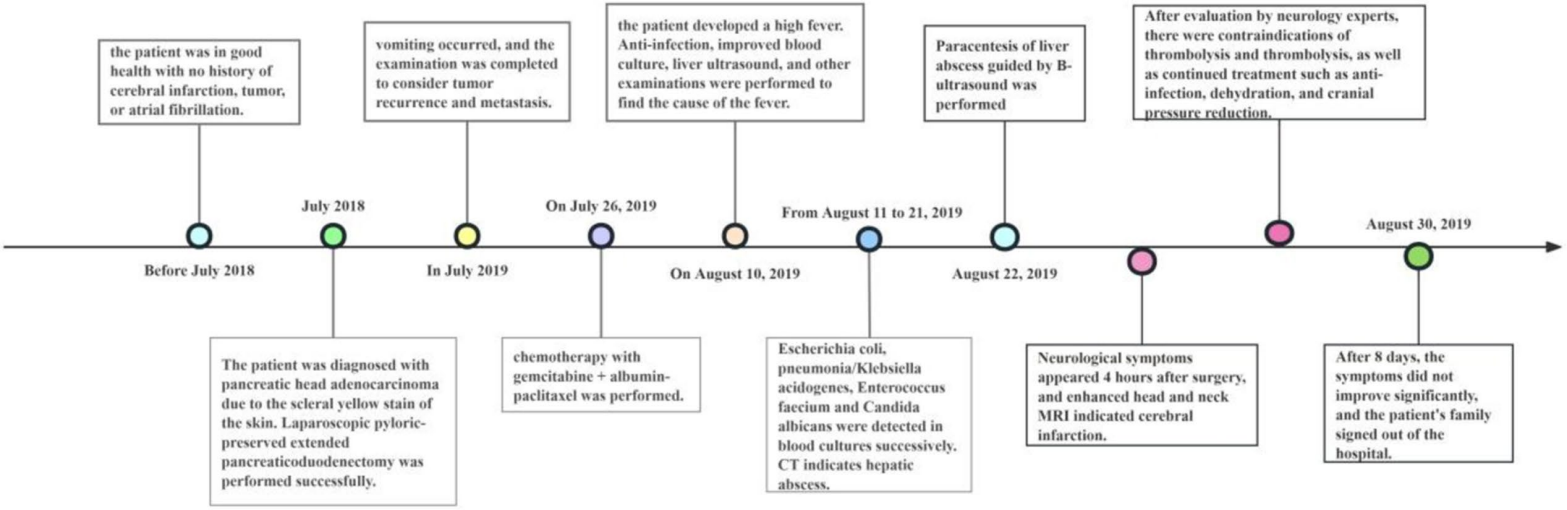
Timeline of the episode of care.

The patient, a 50-year-old woman, was diagnosed with adenocarcinoma of the pancreatic head one year prior and underwent laparoscopic pylorus-preserving pancreaticoduodenectomy. Twenty days before admission, she presented with vomiting. Past medical history: the patient has not had a brain infarction and denies a history of atrial fibrillation or hyperlipidemia. Family history: no family history of tumor, brain infarction, coronary heart disease, etc., and nothing special in the rest. The patient has a physical examination every 3–5 years with no significant abnormal findings. A follow-up abdominal CT suggested tumor recurrence and metastasis near the portal vein main trunk, pancreatic head, and mesentery. She was treated with a chemotherapy regimen including Gemcitabine and albumin-bound paclitaxel. Post-chemotherapy, she developed bleeding, fever, and chills. Laboratory tests revealed anemia, thrombocytopenia, leukopenia, elevated C-reactive protein, and interleukin-6 levels. Blood cultures identified multiple pathogens, and a CT scan indicated a liver abscess (14 × 10 cm), as shown in Figure 1. The patient received various antibiotics and underwent ultrasound-guided liver abscess drainage, yielding over 1,000 mL of pus infected with *Escherichia coli*. Shortly after, she experienced respiratory distress and was treated with oxygen therapy, methylprednisolone, and aminophylline. She then developed right-side limb weakness and left-upper limb fatigue. A head and neck MRI indicated acute brain infarction, as shown in Figure 2. Due to contraindications for thrombolysis or thrombectomy, she continued to receive anti-infection, diuretic, and mannitol treatment. Her condition worsened, resulting in incontinence, mixed aphasia, and decreased right-side sensation. Despite treatment, her symptoms did not improve, and she was discharged at the family’s request. The timeline of the episode of care is showed in Figure 3.

## Discussion

Studies (3) have reported that *Klebsiella pneumoniae* and *Escherichia coli* are the primary pathogens causing pyogenic liver abscess (PLA). PLA is often caused by biliary system obstruction, portal vein reflux obstruction, or systemic bacteremia, and its onset is rapid and typically accompanied by fever and chills. It easily affects organs outside the liver and, in severe cases, can lead to septic shock and multiple organ failure. The incidence of PLA is higher in Asian countries, with about 12–18 cases per 100,000 people (4), and recent studies in developed countries report a mortality rate of 2.6 to 9.6% (5–7). Patients with compromised immune function have a significantly higher poor prognosis rate than those with normal immune function, as they tend to have more severe infections and worse nutritional status when PLA occurs, thus requiring more aggressive treatment strategies. In this case, the patient had a malignant tumor and experienced rapid infection progression due to immunosuppression following chemotherapy, leading to leukopenia and anemia (8). Early blood cultures during the fever indicated infections by multiple pathogens, including *Escherichia coli*, *Klebsiella pneumoniae*, *Enterococcus faecium*, and *Candida albicans*, and a CT scan suggested a liver abscess, which was confirmed by pus culture from the drainage fluid. Anti-infection measures and early aggressive drainage are the primary treatment methods for bacterial liver abscesses. Abscesses smaller than 5 cm are recommended to be treated with aspiration, which can be repeated if necessary (9). In contrast, catheter drainage is recommended for abscesses larger than 5 cm, as it provides continuous drainage, removes thick pus due to its wider diameter, and prevents re-accumulation. Regarding treatment efficacy, ultrasound-guided catheter drainage is safe, has fewer complications, a higher success rate, and reduces the time needed to achieve clinical relief compared to pus aspiration, making it a preferable method (10, 11).

Complications of liver abscess drainage include abscess rupture and intraperitoneal dissemination, but short-term pulmonary or cerebral embolism post-drainage has not been reported. The patient had no history of brain infarction and no high-risk factors such as atrial fibrillation and hyperlipidemia. Therefore, we analyzed the possible causes of this acute brain infarction: bacterial emboli may enter the bloodstream and spread to cerebral vessels, causing cerebral infarction; the patient’s previous diagnosis of pancreatic head adenocarcinoma and abdominal CT findings suggested the possibility of multiple metastases within the liver, and the abscess drainage might have caused cancer emboli to disseminate through the bloodstream to cerebral vessels, leading to cerebral infarction. Additionally, the rapid drainage of a large volume from the patient’s substantial liver abscess could have caused a swift reduction in intracavitary pressure and volume, potentially leading to the collapse of the abscess wall, causing localized damage, and resulting in distant dissemination through blood-borne pus emboli, thereby causing pulmonary embolism and cerebral embolism.

## Conclusion

In summary, we report a rare case of cerebral embolism caused by the drainage of a bacterial liver abscess, highlighting the potential for hematogenous spread and distant organ embolism due to rapid drainage of a large liver abscess.

## Data Availability

The raw data supporting the conclusions of this article will be made available by the authors, without undue reservation.
