# Correction: Case Report: Brain infarction following percutaneous drainage of a liver abscess post-chemotherapy for pancreatic head cancer

**DOI:** 10.3389/fmed.2025.1751397

**Published:** 2025-12-11

**Authors:** Wan-qiu Deng, Bo Wei, Zheng-yan Li, Kai Liu

**Affiliations:** 1 Center of Infectious Diseases, West China Hospital, Sichuan University, Chengdu, China; 2 Department of Gastroenterology, West China Hospital, Sichuan University, Chengdu, China; 3 Department of Radiology, West China Hospital, Sichuan University, Chengdu, China

**Keywords:** chemotherapy, liver abscess, percutaneous drainage, brain infarction, pancreatic head cancer

The funder Key Research and Development Program of Sichuan Province, Grant No. 2023YFS0114 to Bo Wei was erroneously omitted.

The correct **Funding** statement is appears below.

The author(s) declared that financial support was received for this work and/or its publication. This work was supported by the Key Research and Development Program of Sichuan Province (grant no. 2023YFS0114 to Bo Wei).

The original version of this article has been updated.

