# Supplementary material for: Case report: Brain infarction following percutaneous drainage of a liver abscess post-chemotherapy for pancreatic head cancer
Source: Front Med (Lausanne). 2024 Dec 11;11:1453533. doi: 10.3389/fmed.2024.1453533 (PMC11670067; doi:10.3389/fmed.2024.1453533)
Supplement: Supplementary file 1 [file Data_Sheet_1.PDF]

CARE Checklist of information to include when writing a case report

(cc) BY-NC-ND

Topic	Item	Checklist item description	Reported on Line
Title	1	The diagnosis or intervention of primary focus followed by the words "case report"	______
Key Words	2	2 to 5 key words that identify diagnoses or interventions in this case report, including "case report"	______
Abstract (no references)	3a	Introduction: What is unique about this case and what does it add to the scientific literature?	______
	3b	Main symptoms and/or important clinical findings	______
	3c	The main diagnoses, therapeutic interventions, and outcomes	______
	3d	Conclusion—What is the main "take-away" lesson(s) from this case?	______
Introduction	4	One or two paragraphs summarizing why this case is unique (**may include references**)	______
Patient Information	5a	De-identified patient specific information.	______
	5b	Primary concerns and symptoms of the patient.	______
	5c	Medical, family, and psycho-social history including relevant genetic information	______
	5d	Relevant past interventions withoutcomes	______
Clinical Findings	6	Describe significant physical examination (PE) and important clinical findings.	______
Timeline	7	Historical and current information from this episode of care organized as a timeline	______
Diagnostic Assessment	8a	Diagnostic testing (such as PE, laboratory testing, imaging, surveys).	______
	8b	Diagnostic challenges (such as access to testing, financial, or cultural)	______
	8c	Diagnosis (including other diagnoses considered)	______
	8d	Prognosis (such as staging in oncology) where applicable	______
Therapeutic Intervention	9a	Types of therapeutic intervention (such as pharmacologic, surgical, preventive, self-care)	______
	9b	Administration of therapeutic intervention (such as dosage, strength, duration)	______
	9c	Changes in therapeutic intervention (with rationale)	______
Follow-up and Outcomes	10a	Clinician and patient-assessed outcomes (if available)	______
	10b	Important follow-up diagnostic and other test results	______
	10c	Intervention adherence and tolerability (How was this assessed?)	______
	10d	Adverse and unanticipated events	______
Discussion	11a	A scientific discussion of the strengths AND limitations associated with this case report	______
	11b	Discussion of the relevant medical literature **with references**.	______
	11c	The scientific rationale for any conclusions (including assessment of possible causes)	______
	11d	The primary "take-away" lessons of this case report (without references) in a one paragraph conclusion	______
Patient Perspective	12	The patient should share their perspective in one to two paragraphs on the treatment(s) they received	______
Informed Consent	13	Did the patient give informed consent? Please provide if requested	**Yes** √ **No** ☐
